# Exosomal miRNA Profiling in Vitreous Humor in Proliferative Diabetic Retinopathy

**DOI:** 10.3390/cells12010123

**Published:** 2022-12-28

**Authors:** Agnieszka Kot, Radoslaw Kaczmarek

**Affiliations:** Department of Ophthalmology, Wroclaw Medical University, 50-556 Wroclaw, Poland

**Keywords:** miRNA, vitreous, PDR, exosomes

## Abstract

MicroRNAs (miRNAs) are small noncoding RNAs which mediate some of the pathological mechanisms of diabetic retinopathy. The aim of this study was to identify differentially expressed miRNAs in the vitreal exosomes of proliferative diabetic retinopathy (PDR) patients and non-diabetic controls. Exosomes were extracted from the vitreous samples of 10 PDR patients and 10 controls. The expression of 372 miRNAs was determined using a quantitative polymerase chain reaction (qPCR) panel. We have demonstrated a significant dysregulation in 26 miRNAs. The most remarkable findings include a profound attenuation of the miR-125 family, as well as enhanced miR-21-5p expression in the diabetic samples. We also showed the downregulation of miR-204-5p and the upregulation of let-7g in PDR compared to the controls. This study identified miR-125 and miR-21 as potential targets for further functional analysis regarding their putative role in the pathogenesis of PDR.

## 1. Introduction

Diabetic retinopathy (DR) is a leading and growing cause of blindness throughout the world. Proliferative diabetic retinopathy (PDR) represents the most severe form of the disease, characterized by the hallmark feature of retinal neovascularization. Extensive fibrovascular traction can lead to tractional retinal detachment (TRD) which is a major cause of vision loss in PDR and one of the most challenging conditions that vitreoretinal surgeons face. Current management strategies for DR include anti-vascular endothelial growth factor (VEGF) therapy, vitrectomy, and retinal photocoagulation. However, these treatments are targeted to the advanced stages of the disease, have some adverse effects, and may prove ineffective in a considerable number of patients which demands further research into new therapeutic targets and approaches [1]. Recently, a novel type of RNA, microRNA (miRNAs), has been implicated in the pathogenesis of DR. miRNAs are a class of small non-coding RNAs that regulate gene expression at the post-transcriptional level by either degrading or blocking the translation of messenger RNA (mRNA) targets. Several studies have highlighted the crucial role of miRNAs in the regulation of key processes in the development of DR, including angiogenesis and inflammation [2]. miRNA profiling in the vitreous of diabetic patients provides a valuable insight into the complex regulation and expression of genes that is occurring in DR and could lead to novel diagnostic tools as well as therapies to prevent and reverse vision loss for these patients [3].

The use of extracellular vesicles (EV), specifically exosomes, as carriers of miRNA in extracellular spaces has been well demonstrated. Several studies have shown that exosomes provide a protective and enriched source of miRNA for biomarker profiling compared to non-exosomal, cell-free, or whole unfractionated samples [4]. This is of great importance in samples with low miRNA content such as the vitreous. In addition, the exosome content reflects the metabolic status of parental cells and provides valuable information regarding intercellular communication [5].

Thus, the aim of this study was to identify differentially expressed miRNAs in the exosomes that were extracted from the vitreous of PDR patients that were operated on for TRD compared to non-diabetic controls to improve our understanding of the mechanisms leading to PDR.

## 2. Materials and Methods

### 2.1. Patient Selection

This comparative pilot study included twenty patients who underwent pars plana vitrectomy (ppV) in the Ophthalmology Clinic of Wroclaw Medical University, Poland. The study group consisted of ten patients with PDR who developed extensive fibrovascular proliferations and required surgery for TRD. Both patients with Type 1 and Type 2 diabetes were included in the study. All PDR patients received an intravitreal anti-VEGF injection several (2–7) days prior to surgery. Due to ethical reasons concerning possible complications following an unnecessary surgical intervention, healthy subjects could not be enrolled in the study as a control group. We, therefore, used vitreous from ten patients with a non-vascular, non-inflammatory disease—macular hole (MH) to serve as matched controls. To minimize the effects of blood mixture and other ocular or systemic disorders on miRNA expression, we introduced the following exclusion criteria for both groups: vitreous hemorrhage, glaucoma, retinal tear or rhegmatogenous retinal detachment, uveitis, previous vitrectomy or scleral buckling, history of eye trauma, systemic autoimmune diseases, and cardiac and hepatic failure. We also excluded patients with diabetes from the control group.

### 2.2. Acquisition of Vitreous Humor Samples

The vitreous samples were collected during a 3-port 23-gauge pars plana vitrectomy, before starting the infusion. Vitreous specimens were extracted from the core of the vitreous cavity into a syringe using a three-way tap, aliquoted in a 2-mL RNAse-free container, and immediately stored at −80 °C until further analysis. The samples were then transported on dry ice to the Qiagen core laboratory in Germany where RNA isolation and miRNA profiling were conducted.

### 2.3. Isolation of Exosomal Ribonucleic Acid (RNA)

Frozen vitreous was thawed on ice and centrifuged at 3000× *g* for 5 min. Exosomes were precipitated from the supernatants using miRCURY Exosome Isolation Kit—Cells, Urine and CSF (Qiagen, Hilden, Germany), according to manufacturer’s instructions. Total RNA was extracted from the exosomes using miRNeasy Mini Kit (Qiagen, Hilden, Germany), following the manufacturer’s instructions.

### 2.4. miRNA Expression Profiling

A total of 10 μL RNA was reverse transcribed in 50 μL reactions using the miRCURY LNA RT Kit (Qiagen, Hilden, Germany). Complementary deoxyribonucleic acid (cDNA) was diluted 50× and assayed in 10 μL polymerase chain reactions (PCR) according to the protocol for miRCURY LNA miRNA PCR (Qiagen, Hilden, Germany). The expression level of 372 miRNAs was assayed once by quantitative PCR (qPCR) on the miRNA Ready-to-Use PCR, Human panel I (Qiagen, Hilden, Germany; see Supplementary Table S1 for the list of assays) using miRCURY LNA SYBR Green master mix (Qiagen, Hilden, Germany). Negative controls excluding template from the reverse transcription reaction was performed and profiled similar to the samples. The amplification was performed in a LightCycler^®^ 480 Real-Time PCR System (Roche, Basel, Switzerland) in 384 well plates. Fluorescence data were converted into quantification cycle (Cq).

### 2.5. Data Quality Control

RNA spike-in kit (Qiagen, Hilden, Germany) for quality control of the RNA isolation and cDNA synthesis was applied. The RNA isolation controls (UniSp2, UniSp4, and UniSp5) were added to the purification to detect any differences in the extraction efficiency. The cDNA synthesis control (UniSp6) was added in the reverse transcription reaction, giving the opportunity to evaluate the RT reaction. In addition to this, a DNA spike-in (UniSp3) was present on all the panels to rule out inhibition at the qPCR level.

Since neovascularization is a hallmark of PDR, we ascertained that the samples were not contaminated by erythrocyte-derived miRNAs and hemolysis. This was determined by comparing the expression of erythrocyte-enriched miR-451 to the expression of miR-23a which is unaffected by hemolysis.

### 2.6. Data Analysis

The amplification curves were analyzed using the Roche LC software (Roche, Basel, Switzerland), both for the determination of Cq (by the 2nd derivative method) and for melting curve analysis. The amplification efficiency was calculated using algorithms that were similar to the LinReg software. All of the assays were inspected for distinct melting curves and the primer melting temperature was checked to be within known specifications for the assay. Furthermore, the assays had be detected with 5 Cq less than the negative control, and with Cq < 37 to be included in the data analysis. Data that did not pass these criteria were omitted from any further analysis. Since reference genes are unknown for vitreous humour, normalization was performed based on the average of the assays that were detected in all the samples (13 assays). The NormFinder (Version 0.953) algorithm was used as an Excel add-in (available online at http://moma.dk/normfinder-software, accessed on 9 November 2022).

To identify biologically relevant miRNA expression changes between patients with PDR and the control group, the standard approach was employed using a *t*-test and a cut-off of *p*-value < 0.05 as the primary criterion followed by Benjamini–Hochberg correction for false positive errors at a significance level of 0.05.

## 3. Results

### 3.1. Patients’ Characteristics

The patient demographics are provided in Table 1. Vitreous humor samples were extracted from 20 eyes during surgery: 10 eyes with PDR complicated by TRD (mean age 53; range 26–76) and 10 eyes that were affected by MH which served as the control group (mean age 68; range 53–78). A total of 10 patients (50%) were male and 10 (50%) were female. Among the PDR group, seven subjects were diagnosed with Type 2 diabetes and three with Type 1 diabetes. The majority of diabetic cases (8) were insulin-dependent and all patients had poor diabetes control (mean glycated haemoglobin level = 9.7).

### 3.2. Number of Detected miRNAs

Using qPCR technology, we determined the expression of 372 miRNAs in the exosomes that were extracted from the vitreous humor from 10 patients with PDR and 10 controls. The panel profiling detected the presence of 317 different miRNAs. On average, there were 99 miRNAs per sample. A total of 13 miRNAs were detected in all the samples and the global mean of those 13 assays was used for normalization. The stability of the average of 13 miRNAs was higher than any single miRNA in the dataset as measured by the NormFinder software. A total of 99 miRNAs were regularly expressed, being defined as miRNAs that are detectable in at least 70% of all the samples. The most abundant miRNAs in the entire cohort were: miR-98-5p, miR-30e-3p, and miR-375. No significant hemolysis was detected.

### 3.3. Heat Map and Unsupervised Clustering

**Figure 1:**
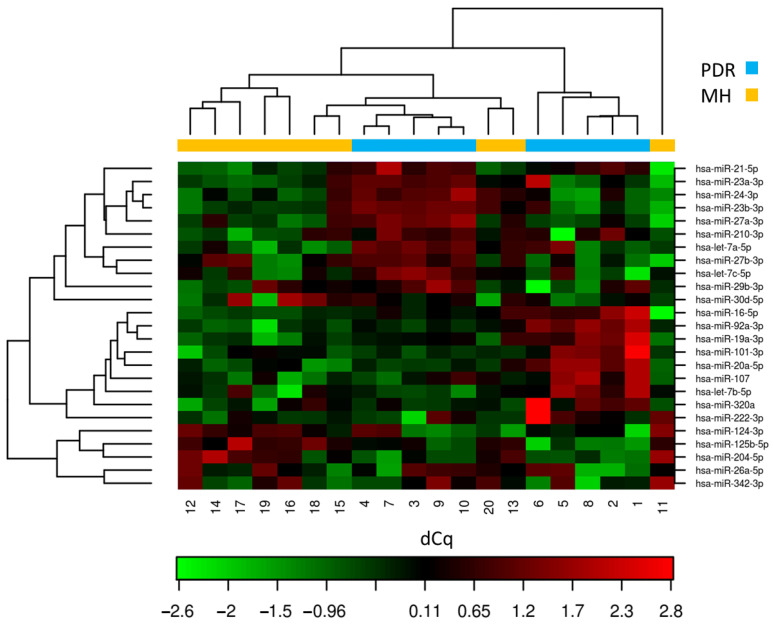
Heat map and the result of the two-way hierarchical clustering of miRNAs and samples.

The normalized Cq (dCq) values of the top 25 most differentially expressed miRNAs are shown in Figure 1. The heat map illustrates the normalized Cq values across the top 25 most differentially expressed miRNAs. The miRNA clustering tree is shown on the left and the samples’ clustering tree on the top of the graph. There was no significant difference in the miRNA expression pattern between patients with Type 1 diabetes and those with Type 2 diabetes.

### 3.4. Differentially Expressed miRNAs in PDR

**Figure 2:**
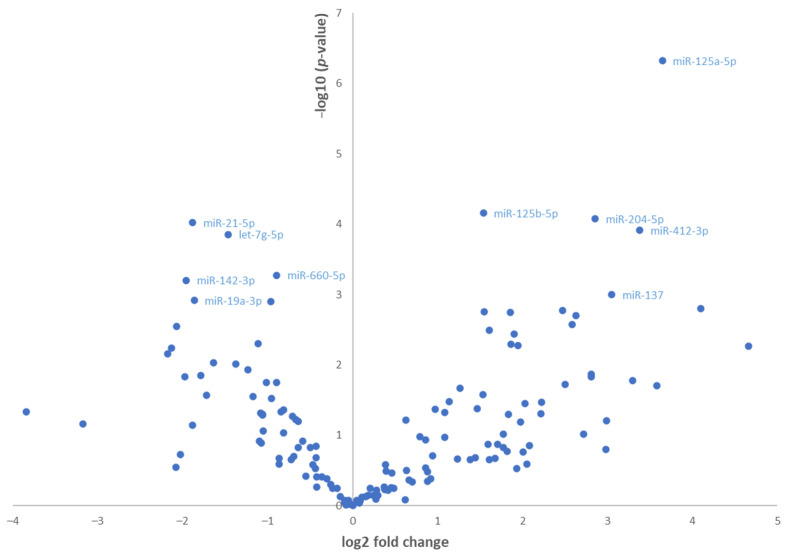
Volcano plot demonstrating the relationship between the *p*-values and the fold change of miRNA expression (dCq) between PDR and the control group. The labels indicate the ten most differentially expressed miRNAs.

When comparing the PDR group to the control group using a t-test, 54 miRNAs were found to be differentially expressed using a cut-off of *p*-value < 0.05. A total of 26 of these passed a Benjamini–Hochberg correction for false positive errors. There were 16 miRNAs that were downregulated (miR-125a-5p, miR-125b-5p, miR-204-5p, miR-412-3p, miR-137, miR-361-3p, miR-211-3p, miR-9-3p, miR-30e-3p, miR-375, miR-9-5p, miR-30a-5p, miR-328-3p, miR-345-5p, miR-100-5p, and miR-543-3p) while 10 miRNAs were upregulated (miR-21-5p, let-7g-5p, miR-660-5p, miR-142-3p, miR-19a-3p, miR-142-5p, miR-15a-5p, miR-103a-3p, miR-92a-3p, and miR-16-5p). The most dysregulated miRNA in the PDR group was miR-125a-5p (fold change = 13, *p*-value after adjustment for false positive errors = 0.000071). There was also a marked overexpression of miR-21-5p and let-7g-5p in the PDR group, as illustrated by the volcano plot in Figure 2.

### 3.5. Targets of Differentially Expressed miRNAs in PDR

To elucidate the possible role of the most dysregulated miRNAs, we analyzed the experimentally validated targets of these miRNAs. The biological functions of the top six most differentially expressed miRNAs which displayed large-magnitude changes that were also statistically significant were annotated by miRTarBase (available online: https://mirtarbase.cuhk.edu.cn, accessed on 9 November 2022) and Online Mendelian Inheritance in Man (OMIM; available online: https://omim.org, accessed on 9 November 2022) database searches and are listed in Table 2 [6]. Focus was on the genes that are known to play a role in PDR and on proteins that were shown to be dysregulated in the vitreous of PDR patients [7,8]. We found strong experimental evidence indicating that the miR-125 family, miR-204-5p, miR-21-5p, miR-41-3p, and let-7g-5p 2 regulate the mechanisms leading to the development of fibrovascular membranes in PDR, including angiogenesis, inflammation, cell adhesion, proliferation and differentiation, as well as extracellular matrix remodeling.

## 4. Discussion

In the current study, we performed large-scale miRNA profiling using a qPCR panel to determine the miRNA expression pattern in the vitreous of PDR patients compared with non-diabetic controls. We have demonstrated a significant dysregulation in 26 miRNAs. The most remarkable results include a profound attenuation of the miR-125 family, as well as enhanced miR-21-5p expression in the diabetic samples. Other findings include the downregulation of miR-204-5p and the upregulation of let-7g in PDR compared to the controls.

A growing body of evidence suggests the pivotal role of miR-21 overexpression in the occurrence of various diabetic complications, including diabetic cardiomyopathy, nephropathy, neuropathy, and DR [9]. In line with this, we showed that miR-21 is upregulated in the vitreous of PDR patients. In retinal and endothelial cells, miR-21 regulates multiple signaling pathways and can simultaneously target various angiogenic factors, including transforming growth factor β (TGF-β) and VEGF [10,11,12]. miR-21 was proposed as a diagnostic and prognostic marker for DR. There are three separate research groups that have found a positive correlation between miR-21 overexpression in the serum of diabetic patients and the severity of DR [13,14,15]. Increased levels of miR-21 have also been reported in the vitreous of patients with fibroproliferative disorders (PDR and proliferative vitreoretinopathy) [16]. A recent study showed that miR-21-3p upregulation promoted pericyte migration and tube formation thus reflecting its role in angiogenic sprouting [17]. Furthermore, miR-21 antagonists have demonstrated great potential in counteracting the high glucose-induced proliferation and angiogenesis of human retinal endothelial cells [11,12]. In addition, the intravitreal injection of an miR-21 inhibitor ameliorated inflammation and reduced microvascular damage in the retina of leptin receptor-deficient (db/db) mice which are used as a genetic model of Type 2 diabetes [18]. Together, the above results suggest the putative role of miR-21 as a therapeutic target and biomarker in PDR.

A dramatic attenuation of miR-125 family expression in the vitreous of PDR patients has been demonstrated in the present study. The miR-125 family has been implicated in modulation of angiogenesis and VEGF signaling pathway in a variety of vascular diseases, warranting further research into its role in the pathogenesis of PDR [19]. Our findings concur well with the experimental data from in vivo rodent models of Type 2 diabetes. A significant downregulation of miR-125a-5p was reported in the retina of streptozotocin (STZ)-induced diabetic rats. Functional analysis revealed that miR-125a-5p regulates the macrophage-mediated vascular integrity and that transfection with an miR-125a-5p mimic inhibits the recruitment of macrophages into inflamed retina resulting in significantly attenuated vascular leakage [20]. The second member of the miR-125 family, miR-125b-5p, is among the most abundantly expressed miRNAs in retinal pigment epithelium (RPE) cells. It is crucial for the maturation and differentiation of RPE cells [21]. High-glucose conditions promote the epithelial-mesenchymal transition (EMT) of RPE cells which is believed to be the key for the development of fibroproliferative disorders, including PDR [22]. Recent evidence indicates that miR-125b-5p supplementation has a protective effect on the maintenance of RPE cell morphology and function and counteracts the hyperglycemia-/hypoxia-induced RPE barrier breakdown [23].

The present study revealed a significant upregulation of let-7g in the vitreous of diabetic samples. In humans, the let-7 family is composed of nine mature let-7 miRNAs that are likely to have functionally redundant roles [24]. The let-7 family regulates various aspects of peripheral glucose metabolism but there are limited data about its involvement in the pathogenesis of PDR [25]. Zhou et al. established a causative role of let-7 in nonproliferative diabetic retinopathy but a repressive function of let-7 in pathological angiogenesis [26]. Overall, further studies are needed to elucidate the involvement of let-7 in PDR.

We found a marked downregulation of miR-204-5p in the vitreous of PDR patients. This is in line with previous experimental data showing that miR-204 expression levels were substantially lower in the retinal tissues in diabetic retinopathy model rats compared to the controls. Moreover, transfection with an miR-204 mimic inhibited the inflammation and cell apoptosis in Sprague-Dawley rats by upregulating B-cell lymphoma 2 (Bcl-2) and sirtuin 1 (SIRT1) [27]. However, other groups reported contradictory results, indicating that miR-204-5p was significantly upregulated in the retina tissue of STZ-induced diabetic rats [28,29]. Thus, further research into the targets of miR-204-5p is required.

To date, limited data are available regarding miRNA expression in the vitreous of PDR patients and there is little consistency between the studies that have been published so far. The present findings corroborate to some extent with previous research but a direct comparison is difficult due to heterogeneity in the populations that have been studied. Some authors analyzed samples that were obtained from patients undergoing surgery due to vitreous hemorrhage, others treated a dense or any vitreous bleeding at all as an exclusion criterion. It is generally accepted that blood mixture should be avoided in miRNA profiling experiments, since cellular fraction and hemolysis will also contribute miRNAs which may bias the analysis [30]. In addition, pre-treatment with intravitreal anti-VEGF injections may influence miRNA expression patterns [31,32]. Other sources of inter-study variability involve both the extraction methodology and the analysis platform that was employed [33]. Moreover, correctly measuring and interpreting miRNA expression in the vitreous is challenging due to the lack of valid internal controls. A method that is widely used for normalization is the addition of synthetic spike-in miRNAs, mainly Caenorhabditis elegans miRNAs (miR-39-3p) or to the endogenous small nuclear RNA U6 although there is no consensus at present whether this is an effective strategy to normalize circulating miRNA levels [34]. In our study, normalization was performed based on the average of the assays that were detected in all the samples as this provides an effective benchmark for qPCR studies involving numerous assays [35].

The strengths of our study include the use of the most specific and sensitive of miRNA profiling platforms available—qPCR. In contrast to hybridization-based methods, it is highly reproducible and allows for accurate quantification, even in samples with low RNA content, such as the vitreous [33]. It should be noted that in some studies, conflicting results were obtained during discovery and validation phases using different miRNA profiling platforms. For example, in a recent study by Solis-Vivanco, the miRNAs that were chosen for validation based on the TaqMan Low Density Arrays were not even detected with qPCR in most of the samples [36]. This finding highlights that microarray analysis should be interpreted with caution and always followed by qPCR validation.

Table 3 presents the studies assessing miRNA expression in vitreous humor in PDR. As mentioned above, there is a striking inconsistency in the results that have been published by different groups. However, there was some overlap between our data and previous reports. Our experiments are in line with a previous study by Smit-McBride et al., showing a marked upregulation of let-7 family members in the vitreous of PDR patients. They also found that let-7 expression was positively correlated with the severity of DR [37]. Similar findings regarding let-7 upregulation and miR-125-5p downregulation in PDR samples were reported by Guo et al. in the next generation sequencing (NGS) screening phase although a validation with qPCR was not performed [32,38]. We were also able to confirm the overexpression of miR-15a, miR142-3p, and miR-19a in the vitreous of diabetic patients, as demonstrated in the literature [31,39,40].

The present study was, to the best of our knowledge, the first one to provide a characterization of miRNA expression in the exosomes of the vitreous samples from PDR patients. Exosomes are a subtype of small extracellular vesicle (EV), formed by an endosomal route and released into extracellular space by all cell types [43]. They have been found in plasma, urine, semen, saliva, and other body fluids and are also abundant in the vitreous. It is not clear which types of cells are the primary source of exosomes in the vitreous but the main candidates include Müller cells and RPE cells [44,45,46]. The cargo of exosomes consists of lipids, proteins, and nucleic acids, including miRNAs. In particular, this exosome-mediated transfer of miRNA has been proposed as a novel mechanism for intercellular communication. Several studies have highlighted the role of exosomes in the modulation of angiogenesis, immunologic response, cell migration, and invasiveness [5]. In the context of diabetic retinopathy, it has been demonstrated that EVs that are derived from mesenchymal stem cells cultured in diabetic-like conditions enter pericytes, cause their detachment and migration, and stimulate angiogenesis [47]. Furthermore, EVs that are extracted from plasma of diabetic retinopathy patients were able to induce features of retinopathy in in vitro models of retinal microvasculature [15]. It was also shown that RPE cells secrete massive amounts of exosomes after EMT, and that these exosomes further mediate the EMT cascade in recipient RPE cells. This finding bears significant implications for vitreoproliferative disorders, including PDR [46].

Our study provides valuable information about the miRNA expression profile in the exosomes of the vitreous in PDR compared to non-diabetic controls. To date, only one study has assessed the exosomal miRNA in the vitreous and it enrolled patients with uveal melanoma [45]. Our findings of miR-21-3p overexpression in the vitreal exosomes of PDR patients are supported by a previous study that reported an increased miR-21-3p concentration inside EV in plasma samples from patients with DR [15]. Another study found a specific enrichment of mir-21-5p in EV compared to the total serum miR-21-5p in diabetic children. The authors concluded that, for certain miRNAs, total circulating miRNA levels are distinct from circulating EV miRNA content [48].

To sum up, an increasing body of experimental evidence indicates the possibility that exosomal miRNA in the vitreous may offer predictive, diagnostic, and prognostic information for DR as well as outline a perspective for novel therapeutic targets but further research is needed to confirm this hypothesis.

The limitations of this study include the small sample size and the use of panels with predefined miRNAs for screening. Furthermore, we used vitreous from MH patients as controls because vitreous of healthy individuals was not available for ethical reasons. Future studies encompassing a functional analysis of dysregulated miRNAs are warranted.

## 5. Conclusions

In conclusion, we provided the miRNA expression profile in the exosomes of PDR patients compared to non-diabetic controls. We identified miR-125 and miR-21 as potential targets for further functional analysis regarding their putative role in the pathogenesis of PDR.

## Figures and Tables

**Table 1:** Patient demographics.

No	Age	Age	Sex	Diabetes Type	Insulin Use	Diabetes Duration
1	PDR	63	F	2	yes	10
2	PDR	65	F	2	yes	26
3	PDR	62	M	2	yes	14
4	PDR	76	M	2	no	21
5	PDR	65	M	2	yes	15
6	PDR	46	F	2	no	3
7	PDR	25	M	1	yes	20
8	PDR	63	F	2	yes	12
9	PDR	39	M	1	yes	1
10	PDR	26	F	1	yes	13
11	MH	53	F	-	-	-
12	MH	56	F	-	-	-
13	MH	63	F	-	-	-
14	MH	65	M	-	-	-
15	MH	78	M	-	-	-
16	MH	77	M	-	-	-
17	MH	72	M	-	-	-
18	MH	61	F	-	-	-
19	MH	78	F	-	-	-
20	MH	78	M	-	-	-

Age and diabetes duration from diagnosis to vitrectomy were given in years.

**Table 2:** Experimentally validated miRNA-target interactions of the top six most differentially expressed miRNAs.

miRNA	PDR	Target	Regulated Processes
miR-125a-5p	↓	VEGFA	angiogenesis
		SMAD4	TGF-β signaling
		EGFR CDKN1A LIN28 TP53 MMP-11 TNFα	cell growth and differentiation cell cycle RPE cells differentiation cell cycle, apoptosis ECM remodeling inflammation
miR-125b-5p	↓	BMP1	TGF-β signaling
miR-204-5p		TGFβ1R, TGFβ2R	cell proliferation and differentiation
		interleukin-1B	inflammation
	↓	BCL2, BIRC2	apoptosis
		ezrin vimentin, E-cadherin	adhesion markers of EMT
miR-21-5p		TGFβ1R, TGFβ2R	cell proliferation and differentiation
	↑	BCL2 PTEN TIMP3, RECK	apoptosis cell cycle ECM remodeling
miR-412-3p	↓	TGFβ1R	cell proliferation and differentiation
let-7g-5p	↑	TGFβR1, IGF2BP1 myc, CDKN2A collagen I fibronectin	cell proliferation and differentiation cell cycle ECM remodeling adhesion, ECM remodeling

↓: miRNA was downregulated in PDR samples; ↑: miRNA was upregulated in PDR samples; ECM = extracellular matrix; EMT = epithelial-mesenchymal transition; RPE = retinal pigment epithelium.

**Table 3:** Studies assessing miRNA expression in vitreous humor in PDR.

Title 1	Discovery Phase	Validation Phase	qPCR Normalization	VH	Anti-VEGF IVI	Differentially Expressed miRNAs after Validation	Overlap with Our Study
Solis-Vivanco (2022) [36]	Microarray (*n* = 39)	qPCR for 2 miRNAs (same cohort, *n* = 39)	U6	no	no	none	-
Yang (2022) [41]	NGS (*n* = 8)	qPCR for 8 miRNAs (same cohort, *n* = 8)	U6	yes	no	miR-889-3p, 939-5p, 4775-3p, 411-5p, 369-3p, 181d-5p, 125a-5p upregulation; miR-1469 downregulation in PDR	-
Guo 2021 [38]	NGS (*n* = 10)	qPCR for 3 miRNAs (*n* = 24)	miR-39-3p	no *	no	miR-3184-3p, -24-3p, -197-3p upregulation in PDR	let-7 family upregulation
Guo 2021 [32]	NGS (*n* = 15)	qPCR for 3 miRNAs (*n* = 26)	miR-39-3p	no *	yes vs. no	miR-3184-3p, -24-3p, -197-3p upregulation in PDR	miR-125b-5p downregulation
Friedrich 2020 [31]	qPCR profiling (*n* = 20)	qPCR for 9 miRNAs (*n* = 40)	none	-	yes vs. no	miR-20a-5p, -23b-3p, -142-3p, -185-5p, -326-5p, -362-5p upregulation in PDR	miR-142-3p upregulation
Smit-McBride (2020) [37]	microarray (*n* = 33)	qPCR for 3 miRNAs (same cohort, *n* = 33)	miR-638, -3613 and -4487	no	no	let-7b upregulation in PDR	let-7 family upregulation
Mammadzada 2019 [39]	qPCR profiling—pooled analysis (*n* = 54)	qPCR for 10 miRNAs— single patient analysis (same cohort, *n* = 54)	miR-39-3p	yes	-	miR-19a, -27a upregulation in PDR	miR-19a upregulation
Gomaa 2017 [42]	qPCR for a single miRNA (*n* = 59)	-	U6	yes	-	miR-200 b upregulation in PDR	-
Usui-Ouchi 2016 [16]	microarray (*n* = 6)	qPCR for 3 miRNAs (*n* = 27)	U6	-	-	miR-21 upregulation in PDR and PVR	miR-21 upregulation
Hirota 2014 [40]	qPCR profiling (*n* = 4)	-	global mean	-	no	miR-15a, -320a, -320b, -93, -29a, -423-5p upregulation in PDR	miR-15a upregulation

*—study excluded patients with a dense vitreous bleeding; *n* = number of patients included in each phase of the study; VH = vitreous hemorrhage; IVI—intravitreal injection; PVR—proliferative vitreoretinopathy.

## Data Availability

Not applicable.

## Supplementary Materials

The following supporting information can be downloaded at: https://www.mdpi.com/article/10.3390/cells12010123/s1, Table S1. List of assays.
