# Supplementary material for: Exosomal miRNA Profiling in Vitreous Humor in Proliferative Diabetic Retinopathy
Source: Cells. 2022 Dec 28;12(1):123. doi: 10.3390/cells12010123 (PMC9818905; doi:10.3390/cells12010123)
Supplement: Supplementary file 1 [file cells-12-00123-s001.zip › cells-2057099-supplementary.pdf]

Table S1. List of assays

miRNA name	microRNA target sequence
hsa-miR-7-5p	UGGAAGACUAGUGAUUUUGUUGU
hsa-miR-217	UACUGCAUCAGGAACUGAUUGGA
hsa-miR-337-5p	GAACGGCUUCAUACAGGAGUU
hsa-miR-328-3p	CUGGCCCUCUCUGCCCUUCCGU
hsa-miR-374b-3p	CUUAGCAGGUUGUAUUAUCAUU
hsa-miR-143-3p	UGAGAUGAAGCACUGUAGCUC
hsa-miR-623	AUCCCUUGCAGGGGCUGUUGGGU
hsa-miR-520c-3p	AAAGUGCUUCCUUUUAGAGGGU
hsa-miR-557	GUUUGCACGGGUGGGCCUUGUCU
hsa-miR-218-5p	UUGUGCUUGAUCUAACCAUGU
hsa-miR-136-5p	ACUCCAUUUGUUUUGAUGAUGGA
hsa-miR-127-5p	CUGAAGCUCAGAGGGCUCUGAU
hsa-miR-140-5p	CAGUGGUUUUACCCUAUGGUAG
hsa-miR-31-3p	UGCUAUGCCAACAUAUUGCCAU
hsa-miR-20b-3p	ACUGUAGUAUGGGCACUUCCAG
hsa-miR-325	CCUAGUAGGUGUCCAGUAAGUGU
hsa-miR-509-3-5p	UACUGCAGACGUGGCAAUCAUG
hsa-miR-210-3p	CUGUGCGUGUGACAGCGGCUGA
hsa-miR-199b-5p	CCCAGUGUUUAGACUAUCUGUUC
hsa-miR-194-5p	UGUAACAGCAACUCCAUGUGGA
hsa-let-7g-5p	UGAGGUAGUAGUUUGUACAGUU
hsa-miR-203a	GUGAAAUGUUUAGGACCACUAG
hsa-miR-181a-3p	ACCAUCGACCGUUGAUUGUACC
hsa-miR-137	UUAUUGCUUAAGAAUACGCGUAG
hsa-miR-551b-3p	GCGACCCAUACUUGGUUUCAG
hsa-miR-524-3p	GAAGGCGCUUCCCUUUGGAGU
hsa-miR-486-5p	UCCUGUACUGAGCUGCCCCGAG
hsa-miR-329-3p	AACACACCUGGUUAACCUCUUU
hsa-miR-487b-3p	AAUCGUACAGGGUCAUCCACUU
hsa-miR-138-5p	AGCUGGUGUUGUGAAUCAGGCCG
hsa-miR-191-5p	CAACGGAAUCCCAAAAGCAGCUG
mmu-miR-378a-3p	ACUGGACUUGGAGUCAGAAGG
hsa-miR-103a-3p	AGCAGCAUUGUACAGGGCUAUGA
hsa-miR-890	UACUUGGAAAGGCAUCAGUUG
hsa-miR-423-5p	UGAGGGGCAGAGAGCGAGACUUU
hsa-miR-221-3p	AGCUACAUUGUCUGCUGGGUUUC
hsa-miR-301b	CAGUGCAAUGAUAUUGUCAAAGC
hsa-miR-550a-5p	AGUGCCUGAGGGAGUAAGAGCCC
hsa-miR-532-5p	CAUGCCUUGAGUGUAGGACCGU
hsa-miR-99a-5p	AACCCGUAGAUCCGAUCUUGUG
hsa-miR-16-5p	UAGCAGCACGUAAAUAUUGGCG
hsa-miR-98-5p	UGAGGUAGUAAGUUGUAUUGUU
hsa-miR-185-5p	UGGAGAGAAAGGCAGUUCCUGA

hsa-miR-25-3p	CAUUGCACUUGUCUCGGUCUGA
hsa-miR-765	UGGAGGAGAAGGAAGGUGAUG
hsa-miR-24-3p	UGGCUCAGUUCAGCAGGAACAG
hsa-miR-369-5p	AGAUCGACCGUGUUAUAUUCGC
hsa-miR-425-5p	AAUGACACGAUCACUCCCGUUGA
hsa-miR-590-5p	GAGCUUAUUCAUAAAAGUGCAG
hsa-miR-760	CGGCUCUGGGUCUGUGGGGA
hsa-miR-574-3p	CACGCUCAUGCACACACCCACA
hsa-miR-130b-3p	CAGUGCAAUGAUGAAAGGGCAU
hsa-miR-30c-5p	UGUAAACAUCCUACACUCUCAGC
hsa-miR-133b	UUUGGUCCCCUUCAACCAGCUA
hsa-miR-524-5p	CUACAAAGGGAAGCACUUUCUC
hsa-miR-23a-3p	AUCACAUUGCCAGGGAUUUCC
hsa-miR-193b-3p	AACUGGCCCUCAAAGUCCCGCU
hsa-miR-501-5p	AAUCCUUUGUCCCUGGGUGAGA
hsa-miR-518c-5p	UCUCUGGAGGGAAGCACUUUCUG
hsa-miR-130a-3p	CAGUGCAAUGUUAAAAGGGCAU
hsa-miR-933	UGUGCGCAGGGAGACCUCUCCC
hsa-miR-379-5p	UGGUAGACUAUGGAACGUAGG
hsa-miR-452-5p	AACUGUUUGCAGAGGAAACUGA
hsa-miR-589-5p	UGAGAACCACGUCUGCUCUGAG
hsa-miR-141-3p	UAACACUGUCUGGUAAAGAUGG
hsa-miR-342-3p	UCUCACACAGAAAUCGCACCCGU
hsa-miR-668-3p	UGUCACUCGGCUCGGCCCACUAC
hsa-miR-934	UGUCUACUACUGGAGACACUGG
hsa-miR-101-3p	UACAGUACUGUGAUAACUGAA
hsa-miR-539-5p	GGAGAAAUUAUCCUUGGUGUGU
hsa-miR-331-3p	GCCCCUGGGCCUAUCCUAGAA
hsa-miR-499a-5p	UUAAGACUUGCAGUGAUGUUU
hsa-miR-196a-5p	UAGGUAGUUUCAUGUUGUUGGG
hsa-miR-888-5p	UACUCAAAAAGCUGUCAGUCA
hsa-miR-330-3p	GCAAAGCACACGGCCUGCAGAGA
hsa-miR-570-3p	CGAAAACAGCAAUUACCUUUGC
hsa-miR-518c-3p	CAAAGCGCUUCUCUUUAGAGUGU
hsa-miR-200a-3p	UAACACUGUCUGGUAACGAUGU
hsa-miR-188-5p	CAUCCCUUGCAUGGUGGAGGG
hsa-miR-26a-5p	UUCAAGUAAUCCAGGAUAGGCU
hsa-miR-99b-5p	CACCCGUAGAACCGACCUUGCG
hsa-miR-431-5p	UGUCUUGCAGGCCGUCAUGCA
hsa-miR-23b-3p	AUCACAUUGCCAGGGAUUACC
hsa-miR-367-3p	AAUUGCACUUUAGCAAUGGUGA
hsa-miR-505-3p	CGUCAACACUUGCUGGUUUCCU
hsa-miR-18a-5p	UAAGGUGCAUCUAGUGCAGAUAG
hsa-miR-92a-3p	UAUUGCACUUGUCCCGGCCUGU
hsa-miR-500a-5p	UAAUCCUUGCUACCUGGGUGAGA
hsa-miR-887-3p	GUGAACGGGCGCCAUCCCGAGG

hsa-miR-491-3p	CUUAUGCAAGAUUCCCUUCUAC
hsa-miR-423-3p	AGCUCGGUCUGAGGCCCCUCAGU
hsa-miR-126-3p	UCGUACCGUGAGUAAUAAUGCG
hsa-miR-421	AUCAACAGACAUUAAUUGGGCGC
hsa-miR-376b-3p	AUCAUAGAGGAAAAUCCAUGUU
hsa-miR-302c-3p	UAAGUGCUUCCAUGUUUCAGUGG
hsa-miR-625-3p	GACUAUAGAACUUUCCCCCUCA
hsa-miR-339-5p	UCCCUGUCCUCCAGGAGCUCACG
hsa-miR-873-5p	GCAGGAACUUGUGAGUCUCCU
hsa-miR-323a-3p	CACAUUACACGGUCGACCUCU
hsa-miR-181d-5p	AACAUUCAUUGUUGUCGGUGGGU
hsa-miR-125a-5p	UCCCUGAGACCCUUUAACCUGUGA
hsa-miR-129-5p	CUUUUUGCGGUCUGGGCUUGC
hsa-miR-492	AGGACCUGCGGGACAAGAUUCUU
hsa-miR-20a-5p	UAAAGUGCUUAUAGUGCAGGUAG
hsa-miR-374b-5p	AUAUAAUACAACCUGCUAAGUG
hsa-miR-302d-3p	UAAGUGCUUCCAUGUUUGAGUGU
hsa-miR-346	UGUCUGCCCGCAUGCCUGCCUCU
hsa-miR-151a-3p	CUAGACUGAAGCUCCUUGAGG
hsa-miR-493-3p	UGAAGGUCUACUGUGUGCCAGG
hsa-miR-122-5p	UGGAGUGUGACAAUGGUGUUUG
hsa-miR-99a-3p	CAAGCUCGCUUCUAUGGGUCUG
hsa-miR-361-5p	UUAUCAGAAUCUCCAGGGGUAC
hsa-miR-202-3p	AGAGGUAUAGGGCAUGGGAA
hsa-miR-125b-5p	UCCCUGAGACCCUAACUUGUGA
hsa-miR-503-5p	UAGCAGCGGGAACAGUUCUGCAG
hsa-miR-204-5p	UUCCCUUUGUCAUCCUAUGCCU
hsa-miR-30d-5p	UGUAAACAUCCCCGACUGGAAG
hsa-miR-301a-3p	CAGUGCAAUAGUAUUGUCAAAGC
hsa-miR-362-5p	AAUCCUUGGAACCUAGGUGUGAGU
hsa-miR-30b-3p	CUGGGAGGUGGAUGUUUACUUC
hsa-miR-654-5p	UGGUGGGCCGCAGAACAUGUGC
hsa-miR-545-3p	UCAGCAAACAUUUAUUGUGUGC
hsa-miR-29b-2-5p	CUGGUUUCACAUGGUGGCUUAG
hsa-miR-491-5p	AGUGGGGAACCCUUCCAUGAGG
hsa-miR-92b-3p	UAUUGCACUCGUCCCGGCCUCC
hsa-miR-665	ACCAGGAGGCUGAGGCCCCU
hsa-miR-506-3p	UAAGGCACCCUUCUGAGUAGA
hsa-miR-363-3p	AAUUGCACGGUAUCCAUCUGUA
hsa-miR-132-3p	UAACAGUCUACAGCCAUGGUCG
hsa-miR-651-5p	UUUAGGAUAAGCUUGACUUUUG
hsa-miR-628-3p	UCUAGUAAGAGUGGCAGUCGA
hsa-miR-432-5p	UCUUGGAGUAGGUCAUUGGGUGG
hsa-miR-154-3p	AAUCAUACACGGUUGACCUAUU
hsa-miR-27a-3p	UUCACAGUGGCUAAGUUCCGC
hsa-miR-376c-3p	AACAUAGAGGAAAUUCCACGU

hsa-miR-940	AAGGCAGGGCCCCCGCUCCCC
hsa-miR-22-5p	AGUUCUUCAGUGGCAAGCUUUA
hsa-miR-224-5p	CAAGUCACUAGUGGUUCCGUU
hsa-miR-885-5p	UCCAUUACACUACCCUGCCUCU
hsa-miR-320a	AAAAGCUGGGUUGAGAGGGCGA
hsa-miR-18b-5p	UAAGGUGCAUCUAGUGCAGUUAG
hsa-miR-187-3p	UCGUGUCUUGUGUUGCAGCCGG
hsa-miR-516b-5p	AUCUGGAGGUAAGAAGCACUUU
hsa-miR-302c-5p	UUUAACAUGGGGGUACCUGCUG
hsa-miR-548b-3p	CAAGAACCUCAGUUGCUUUUGU
hsa-miR-186-5p	CAAAGAAUUCUCCUUUUGGGCU
hsa-miR-199a-5p	CCCAGUGUUCAGACUACCUGUUC
hsa-miR-155-5p	UUAAUGCUAAUCGUGAUAGGGGU
hsa-miR-107	AGCAGCAUUGUACAGGGCUAUCA
hsa-miR-302b-3p	UAAGUGCUUCCAUGUUUUAGUAG
hsa-miR-662	UCCCACGUUGUGGCCCAGCAG
hsa-miR-519d-3p	CAAAGUGCCUCCCUUUAGAGUG
hsa-miR-485-3p	GUCAUACACGGCUCUCCUCUCU
hsa-miR-200b-3p	UAAUACUGCCUGGUAAUGAUGA
hsa-miR-337-3p	CUCCUAUAUGAUGCCUUUCUUC
hsa-miR-494-3p	UGAAACAUACACGGGAAACCUC
hsa-miR-371a-3p	AAGUGCCGCCAUCUUUUGAGUGU
hsa-miR-637	ACUGGGGGCUUUCGGGCUCUGCGU
hsa-miR-144-3p	UACAGUAUAGAUGAUGUACU
hsa-miR-16-1-3p	CCAGUAUUAACUGUGCUGCUGA
hsa-miR-631	AGACCUGGCCCAGACCUCAGC
hsa-miR-34c-5p	AGGCAGUGUAGUUAGCUGAUUGC
hsa-miR-211-5p	UUCCCUUUGUCAUCCUUCGCCU
hsa-miR-454-3p	UAGUGCAAUAUUGCUUAUAGGGU
hsa-let-7f-5p	UGAGGUAGUAGAUUGUAUAGUU
hsa-miR-30e-5p	UGUAAACAUCCUUGACUGGAAG
hsa-miR-34a-5p	UGGCAGUGUCUUAGCUGGUUGU
hsa-miR-663a	AGGCGGGGCGCCGCGGGACCGC
hsa-miR-518e-3p	AAAGCGCUUCCCUUCAGAGUG
hsa-miR-29b-3p	UAGCACCAUUUGAAAUCAGUGUU
hsa-miR-658	GGCGGAGGGAAGUAGGUCCGUUGGU
hsa-miR-572	GUCCGCUCGGCGGUGGCCCA
hsa-miR-802	CAGUAACAAAGAUUCAUCCUUGU
hsa-miR-521	AACGCACUUCCCUUUAGAGUGU
hsa-miR-433-3p	AUCAUGAUGGGCUCCUCGGUGU
hsa-miR-660-5p	UACCCAUUGCAUAUCGGAGUUG
hsa-let-7c-5p	UGAGGUAGUAGGUUGUAUGGUU
hsa-miR-28-5p	AAGGAGCUCACAGUCUAUUGAG
hsa-miR-324-5p	CGCAUCCCCUAGGGCAUUGGUGU
hsa-miR-219a-5p	UGAUUGUCCAAACGCAAUUCU
hsa-miR-19b-3p	UGUGCAAAUCCAUGCAAAACUGA

hsa-miR-526b-5p	CUCUUGAGGGAAGCACUUUCUGU
hsa-miR-215-5p	AUGACCUAUGAAUUGACAGAC
hsa-miR-30b-5p	UGUAAACAUCCUACACUCAGCU
hsa-miR-184	UGGACGGAGAACUGAUAAGGGU
hsa-miR-422a	ACUGGACUUAGGGUCAGAAGGC
hsa-miR-199a-3p	ACAGUAGUCUGCACAUUGGUUA
hsa-miR-335-5p	UCAAGAGCAAUAACGAAAAAUGU
hsa-miR-519a-3p	AAAGUGCAUCCUUUUAGAGUGU
hsa-miR-21-5p	UAGCUUAUCAGACUGAUGUUGA
hsa-miR-129-2-3p	AAGCCCUUACCCCAAAAAGCAU
hsa-miR-26b-5p	UUCAAGUAAUUCAGGAUAGGU
hsa-miR-214-3p	ACAGCAGGCACAGACAGGCAGU
hsa-miR-32-5p	UAUUGCACAUUACUAAGUUGCA
hsa-miR-324-3p	ACUGCCCCAGGUGCUGCUGG
hsa-miR-488-3p	UUGAAAGGCUAUUUCUUGGUC
hsa-miR-371a-5p	ACUCAAACUGUGGGGGCACU
hsa-miR-455-5p	UAUGUGCCUUUGGACUACAUCG
hsa-miR-891a-5p	UGCAACGAACCUGAGCCACUGA
hsa-miR-549a	UGACAACUAUGGAUGAGCUCU
hsa-miR-205-5p	UCCUUCAUUCCACCGGAGUCUG
hsa-miR-518b	CAAAGCGCUCCCCUUUAGAGGU
hsa-miR-19a-3p	UGUGCAAAUCUAUGCAAAACUGA
hsa-miR-150-5p	UCUCCCAACCCUUGUACCAGUG
hsa-miR-15a-5p	UAGCAGCACAUAAUGGUUUGUG
hsa-let-7d-3p	CUAUACGACCUGCUGCCUUUCU
hsa-miR-608	AGGGGUGGUGUUGGGACAGCUCCGU
hsa-miR-671-5p	AGGAAGCCCUGGAGGGGCUGGAG
hsa-miR-497-5p	CAGCAGCACACUGUGGUUUGU
hsa-miR-877-5p	GUAGAGGAGAUGGCGCAGGG
hsa-miR-187-5p	GGCUACAACACAGGACCCGGGC
hsa-miR-10b-5p	UACCCUGUAGAACCGAAUUUGUG
hsa-let-7i-5p	UGAGGUAGUAGUUUGUGCUGUU
hsa-miR-202-5p	UUCCUAUGCAUAUACUUCUUUG
hsa-miR-652-3p	AAUGGCGCCACUAGGGUUGUG
hsa-miR-126-5p	CAUUAUUACUUUUGGUACGCG
hsa-miR-30e-3p	CUUUCAGUCGGAUGUUUACAGC
hsa-miR-181c-5p	AACAUUCAACCUGUCGGUGAGU
hsa-miR-9-3p	AUAAAGCUAGAUAACCGAAAGU
hsa-miR-548c-3p	CAAAAAUCUCAAUUACUUUUGC
hsa-miR-152-3p	UCAGUGCAUGACAGAACUUGG
hsa-miR-93-5p	CAAAGUGCUGUUCGUGCAGGUAG
hsa-miR-365a-3p	UAAUGCCCCUAAAAAUCCUUAU
hsa-miR-29c-3p	UAGCACCAUUUGAAAUCGGUUA
hsa-miR-372-3p	AAAGUGCUGCGACAUUUGAGCGU
hsa-miR-133a-3p	UUUGGUCCCCUUCAACCAGCUG
hsa-miR-124-3p	UAAGGCACGCGGUGAAUGCC

hsa-miR-190a-5p	UGAUAUGUUUGAUAUAUUAGGU
hsa-miR-302a-3p	UAAGUGCUUCCAUGUUUUGGUGA
hsa-miR-595	GAAGUGUGCCGUGGUGUGUCU
hsa-miR-602	GACACGGGCGACAGCUGCGGCCC
hsa-miR-223-3p	UGUCAGUUUGUCAAAUACCCCA
hsa-miR-627-5p	GUGAGUCUCUAAGAAAAGAGGA
hsa-miR-34b-3p	CAAUCACUAACUCCACUGCCAU
hsa-miR-410-3p	AAUAUAACACAGAUGGCCUGU
hsa-miR-17-5p	CAAAGUGCUUACAGUGCAGGUAG
hsa-miR-376a-3p	AUCAUAGAGGAAAAUCCACGU
hsa-miR-514a-3p	AUUGACACUUCUGUGAGUAGA
hsa-miR-512-5p	CACUCAGCCUUGAGGGCACUUUC
hsa-miR-449a	UGGCAGUGUAUUGUUAGCUGGU
hsa-miR-498	UUUCAAGCCAGGGGGCGUUUUUC
hsa-miR-148b-3p	UCAGUGCAUCACAGAACUUUGU
hsa-miR-127-3p	UCGGAUCCGUCUGAGCUUGGCU
hsa-miR-598-3p	UACGUCAUCGUUGUCAUCGUCA
hsa-miR-96-5p	UUUGGCACUAGCACAUUUUUGCU
hsa-let-7d-5p	AGAGGUAGUAGGUUGCAUAGUU
hsa-miR-135b-5p	UAUGGCUUUUCAUUCCUAUGUGA
hsa-miR-495-3p	AAACAAACAUGGUGCACUUCUU
hsa-miR-299-5p	UGGUUUACCGUCCCACAUACAU
hsa-miR-34c-3p	AAUCACUAACCACACGGCCAGG
hsa-miR-596	AAGCCUGCCCGGCUCCUCGGG
hsa-miR-744-5p	UGCGGGGCUAGGGCUAACAGCA
hsa-miR-145-5p	GUCCAGUUUUCCCAGGAAUCCCU
hsa-miR-622	ACAGUCUGCUGAGGUUGGAGC
hsa-miR-516a-5p	UUCUCGAGGAAAGAAGCACUUUC
hsa-let-7a-5p	UGAGGUAGUAGGUUGUAUAGUU
hsa-miR-96-3p	AAUCAUGUGCAGUGCCAAUAUG
hsa-miR-185-3p	AGGGGCUGGCUUUCCUCUGGUC
hsa-miR-615-3p	UCCGAGCCUGGGUCUCCCUCUU
hsa-miR-128-3p	UCACAGUGAACCGGUCUCUUU
hsa-miR-766-3p	ACUCCAGCCCCACAGCCUCAGC
hsa-miR-206	UGGAAUGUAAGGAAGUGUGUGG
hsa-miR-298	AGCAGAAGCAGGGAGGUUCUCCCA
hsa-miR-193a-5p	UGGGUCUUUGCGGGCGAGAUGA
hsa-miR-449b-5p	AGGCAGUGUAUUGUUAGCUGGC
hsa-miR-520d-5p	CUACAAAGGGAAGCCCUUUC
hsa-miR-192-5p	CUGACCUAUGAAUUGACAGCC
hsa-miR-29a-3p	UAGCACCAUCUGAAAUCGGUUA
hsa-miR-18a-3p	ACUGCCCUAAGUGCUCCUUCUGG
hsa-miR-383-5p	AGAUCAGAAGGUGAUUGUGGCU
hsa-miR-9-5p	UCUUUGGUUAUCUAGCUGUAUGA
hsa-miR-142-5p	CAUAAAGUAGAAAGCACUACU
hsa-miR-363-5p	CGGGUGGAUCACGAUGCAAUUU

hsa-miR-147b	GUGUGCGGAAAUGCUUCUGCUA
hsa-miR-197-3p	UUCACCACCUUCUCCACCCAGC
hsa-miR-597-5p	UGUGUCACUCGAUGACCACUGU
hsa-miR-326	CCUCUGGGCCCUUCCUCCAG
hsa-miR-15b-5p	UAGCAGCACAUCAUGGUUUACA
hsa-miR-105-5p	UCAAAUGCUCAGACUCCUGUGGU
hsa-miR-196b-5p	UAGGUAGUUUCCUGUUGUUGGG
hsa-miR-296-5p	AGGGCCCCCCCUCAAUCCUGU
hsa-miR-20b-5p	CAAAGUGCUCAUAGUGCAGGUAG
hsa-miR-147a	GUGUGUGGAAAUGCUUCUGC
hsa-miR-198	GGUCCAGAGGGGAGAUAGGUUC
hsa-miR-375	UUUGUUCGUUCGGCUCGCGUGA
hsa-miR-517a-3p	AUCGUGCAUCCCUUUAGAGUGU
hsa-miR-361-3p	UCCCCCAGGUGUGAUUCUGAUUU
hsa-miR-21-3p	CAACACCAGUCGAUGGGCUGU
hsa-miR-373-3p	GAAGUGCUUCGAUUUUGGGGUGU
hsa-miR-518f-3p	GAAAGCGCUUCUCUUUAGAGG
hsa-miR-222-3p	AGCUACAUCUGGCUACUGGGU
hsa-miR-617	AGACUUCCCAUUUGAAGGUGGC
hsa-miR-154-5p	UAGGUUAUCCGUGUUGCCUUCG
hsa-miR-708-5p	AAGGAGCUUACAAUCUAGCUGGG
hsa-let-7b-5p	UGAGGUAGUAGGUUGUGUGGUU
hsa-miR-95-3p	UUCAACGGGUAUUUAUUGAGCA
hsa-miR-517c-3p	AUCGUGCAUCCUUUUAGAGUGU
hsa-miR-151a-5p	UCGAGGAGCUCACAGUCUAGU
hsa-miR-502-5p	AUCCUUGCUAUCUGGGUGCUA
hsa-miR-345-5p	GCUGACUCCUAGUCCAGGGCUC
hsa-miR-509-3p	UGAUUGGUACGUCUGUGGGUAG
hsa-miR-134-5p	UGUGACUGGUUGACCAGAGGGG
hsa-miR-382-5p	GAAGUUGUUCGUGGUGGAUUCG
hsa-miR-490-3p	CAACCUGGAGGACUCCAUGCUG
hsa-miR-200c-3p	UAAUACUGCCGGGUAAUGAUGGA
hsa-miR-30a-5p	UGUAAACAUCCUCGACUGGAAG
hsa-miR-181b-5p	AACAUUCAUUGCUGUCGGUGGGU
hsa-miR-33a-5p	GUGCAUUGUAGUUGCAUUGCA
hsa-miR-195-5p	UAGCAGCACAGAAAUAUUGGC
hsa-miR-874-3p	CUGCCCUGGCCCGAGGGACCGA
hsa-miR-135a-5p	UAUGGCUUUUUAUUCCUAUGUGA
hsa-miR-26a-2-3p	CCUAUUCUUGAUUACUUGUUUC
hsa-miR-146b-5p	UGAGAACUGAAUUCCAUAGGCU
hsa-miR-412-3p	ACUUCACCUGGUCCACUAGCCGU
hsa-miR-1	UGGAAUGUAAAGAAGUAUGUAU
hsa-miR-299-3p	UAUGUGGGAUGGUAAACCGCUU
hsa-miR-142-3p	UGUAGUGUUUCCUACUUUAUGGA
hsa-miR-338-3p	UCCAGCAUCAGUGAUUUUGUUG
hsa-miR-584-5p	UUAUGGUUUGCCUGGGACUGAG

hsa-miR-377-3p	AUCACACAAAGGCAACUUUUGU
hsa-miR-216a-5p	UAAUCUCAGCUGGCAACUGUGA
hsa-miR-424-5p	CAGCAGCAAUUCAUGUUUUGAA
hsa-miR-921	CUAGUGAGGGACAGAACCAGGAUUC
hsa-miR-513a-5p	UUCACAGGGAGGUGUCAU
hsa-miR-140-3p	UACCACAGGGUAGAACCACGG
hsa-miR-181a-5p	AACAUUCAACGCUGUCGGUGAGU
hsa-miR-10a-5p	UACCCUGUAGAUCCGAAUUUGUG
hsa-miR-106a-5p	AAAAGUGCUUACAGUGCAGGUAG
hsa-miR-182-5p	UUUGGCAAUGGUAGAACUCACACU
hsa-miR-370-3p	GCCUGCUGGGGUGGAACCUGGU
hsa-miR-576-5p	AUUCUAAUUUCUCCACGUCUUU
hsa-miR-425-3p	AUCGGGAAUGUCGUGUCCGCCC
hsa-miR-450a-5p	UUUUGCGAUGUGUUCCUAAUAU
hsa-miR-411-5p	UAGUAGACCGUAUAGCGUACG
hsa-miR-216b-5p	AAAUCUCUGCAGGCAAAUGUGA
hsa-miR-106b-5p	UAAAGUGCUGACAGUGCAGAU
hsa-miR-22-3p	AAGCUGCCAGUUGAAGAACUGU
hsa-miR-510-5p	UACUCAGGAGAGUGGCAAUCAC
hsa-miR-212-3p	UAACAGUCUCCAGUCACGGCC
hsa-miR-525-5p	CUCCAGAGGGAUGCACUUUCU
hsa-miR-542-5p	UCGGGGAUCAUCAUGUCACGAGA
hsa-miR-576-3p	AAGAUGUGGAAAAAUUGGAAUC
hsa-miR-583	CAAAGAGGAAGGUCCCAUUAC
hsa-miR-483-3p	UCACUCCUCUCCUCCCGUCUU
hsa-miR-582-5p	UUACAGUUGUUCAACCAGUUACU
hsa-miR-183-5p	UAUGGCACUGGUAGAAUUCACU
hsa-miR-33b-5p	GUGCAUUGCUGUUGCAUUGC
hsa-miR-193a-3p	AACUGGCCUACAAAGUCCCAGU
hsa-miR-153-3p	UUGCAUAGUCACAAAAGUGAUC
hsa-let-7e-5p	UGAGGUAGGAGGUUGUAUAGUU
hsa-miR-409-3p	GAAUGUUGCUCGGUGAACCCCU
hsa-miR-100-5p	AACCCGUAGAUCCGAACUUGUG
hsa-miR-629-5p	UGGGUUUACGUUGGGAGAACU
hsa-miR-484	UCAGGCUCAGUCCCCUCCCGAU
hsa-miR-429	UAAUACUGUCUGGUAAAACCGU
hsa-miR-30c-2-3p	CUGGGAGAAGGCUGUUUACUCU
hsa-miR-518a-3p	GAAAGCGCUUCCCUUUGCUGGA
hsa-miR-340-5p	UUAUAAAGCAAUGAGACUGAUU
hsa-miR-508-3p	UGAUUGUAGCCUUUUGGAGUAGA
hsa-miR-381-3p	UAUACAAGGGCAAGCUCUCUGU
hsa-miR-148a-3p	UCAGUGCACUACAGAACUUUGU
hsa-miR-146a-5p	UGAGAACUGAAUUCCAUGGGUU
hsa-miR-139-5p	UCUACAGUGCACGUGUCUCCAGU
hsa-miR-373-5p	ACUCAAAAUGGGGGCGCUUUCC
hsa-miR-149-5p	UCUGGCUCCGUGUCUUCACUCCC

hsa-miR-642a-5p	GUCCCUCUCCAAAUGUGUCUUG
hsa-miR-31-5p	AGGCAAGAUGCUGGCAUAGCU
hsa-miR-451a	AAACCGUUACCAUUACUGAGUU
hsa-miR-620	AUGGAGAUAGAUAUAGAAAU
hsa-miR-27b-3p	UUCACAGUGGCUAAGUUCUGC
hsa-miR-523-3p	GAACGCGCUUCCCUAUAGAGGGU
hsa-miR-374a-5p	UUAUAAUACAACCUGAUAAGUG
